# Consistency, now what?

**DOI:** 10.1186/s13058-017-0869-7

**Published:** 2017-07-21

**Authors:** Mary Beth Terry

**Affiliations:** 1 Department of Epidemiology, Columbia University Mailman School of Public Health, 722 West 168th Street, Room 1611, New York, NY 10032 USA; 2 Herbert Irving Comprehensive Cancer Center, Columbia University Medical Center, New York, NY USA

**Keywords:** Breast cancer, Body size, Adolescence

In epidemiology, inconsistencies are quite common, consistency less so. In this issue of *Breast Cancer Research*, Shawon and colleagues provide more evidence of consistency for the inverse associations between childhood and adolescent body size and breast cancer risk [1]. Consistent with previous literature based on both case-control and cohort studies (e.g., [2–4]), they observe an inverse association between larger body size at age 7 and 18 years and breast cancer risk in over 35,000 women including over 6700 women with breast cancer. Their study, by pooling data from two individual studies, is larger than most allowing greater precision in estimating separate effects by subgroups. The similarity of their findings between both estrogen receptor (ER)-positive and ER-negative breast cancers and in pre- and postmenopausal women suggests that the inverse association with childhood body size and breast cancer risk is applicable across the spectrum of underlying breast cancer risk and not specific to tumor subtypes more commonly seen in young women. The consistency observed in the study subgroups builds on a literature that was already consistent across study designs and with breast cancer risk and two of its strongest risk factors, benign breast disease [5] and mammographic density [6].

Shawon and colleagues [1] use pictograms, previously validated [7] by others with fair to good correlation. In addition, the main comparison in epidemiology, as with Shawon and colleagues, is often between the highest and lowest categories where the error is substantially less than adjacent categories. Pictograms can also be compared with questions about relative comparisons, and Robinson and colleagues reported differences by race in the responses to recalled body size when using absolute categories from pictograms but saw similar inverse associations with adolescent body size when making references to peer group [8]. Inverse associations with breast cancer risk have also been seen for relative comparison (e.g., were you heavier/shorter, taller/lighter than your peers at a particular age) without accompanying pictograms [2]. Importantly, Ahlgren et al. [9] reported inverse associations with breast cancer risk when using school records. Thus, the observed inverse association is robust and consistently seen regardless of the types of measurements.

Epidemiologic inference, particularly with observational studies, can be enhanced by repeated measures. Shawon and colleagues observed a 10% reduction in breast cancer risk for those who reduced a major body size category from 7 to 18 years [1], although only 15% changed a major category of body size. A similar reduction in breast cancer risk from reducing body size categories over adolescence was also seen by Baer et al. [4], but when Baer and colleagues further adjusted for body size category at the time of the first measurement there was no longer any association highlighting the methodologic challenge of making inference about change from recalled data where stability in body size may be partially explained by consistent responses in category. Prospective studies that measure childhood and adolescent body size changes are greatly needed to examine whether changes in growth patterns increase or decrease risk over adolescence.

The consistent inverse association between early-life body size and breast cancer risk is matched by a similarly consistent association between larger adult body size and increased breast cancer risk. Given the positive association between large body size in adulthood and breast cancer as well as many other cancers and chronic diseases, and the fact that adult body size is shaped by growth trajectories much earlier in life [10], the consistent inverse association with larger body size during childhood and adolescence has largely been ignored from a public health perspective. Unraveling the opposing effects of body size on breast cancer risk requires more evidence from in vitro and animal studies as well as prospective studies in humans that can measure changes in breast tissue characteristics. There exists a growing and compelling set of laboratory data supporting the mechanisms between weight gain and metabolically rich environments in increasing breast cancer risk including inflammation and metabolic processes related to cancer risk and changes to epigenetically regulated genes like *BRCA1* [11]. Recently the ability to measure normal changes to breast tissue across adolescence in a non-invasive way has become possible [12]. Interestingly, while increased glucose and nutrients may alter the ability of *BRCA1* to function as a tumor suppressor [11], pre-pubertal estrogen exposure may increase the ability of key breast cancer susceptibility genes to decrease breast cancer risk through cellular differentiation [13]. Thus overweight girls, even though they experience an earlier puberty, may have increased breast cell differentiation from pre-pubertal estrogen exposure converted from androgens in their adipose tissue. Epidemiologic studies have helped to rule out menstrual cycle abnormalities as another hypothesis of why overweight adolescent girls may have a decreased risk of breast cancer [14]. Much still needs to be known about the role of the pre-pubertal environment in shaping breast cancer risk and prospective studies that collect pre-pubertal biospecimens and can follow girls throughout adolescence are needed to examine how hormonal, metabolic, and growth factors relate to changes at the breast tissue level. A key link to explaining the inverse association may be through the growth of dense tissue and changes to the architecture surrounding the dense tissue which develops and rapidly grows throughout adolescence [15].

In addition to measuring childhood and adolescent growth prospectively and using pre-pubertal biospecimens to measure whether biomarkers of the pre-pubertal environment help to explain differences in breast tissue characteristics based on body size, there remains another piece of the puzzle that needs to be completed. Specifically, it is unclear whether the breast cancer risk in girls that are larger in childhood and adolescence and are large at birth and infancy differs from the risk in girls who start small and grow rapidly. The inverse association between adolescent body size and breast cancer risk may not be apparent for the former [3]. Epidemiology in its search for consistency is always at its best when driven by questions that arise from inconsistencies and an appreciation of the fact that exposures that operate in one way at a time in life may work in different ways across the life-course as the organs that affect disease etiology are evolving and developing and changing in form and function.

## Abbreviation

ER: Estrogen receptor
